# The relationship between school meals with thinness and stunting among primary school students, in Meket Wereda, Ethiopia: comparing schools with feeding and non-feeding program

**DOI:** 10.1186/s40795-020-00358-3

**Published:** 2020-08-11

**Authors:** Yeshalem Mulugeta Demilew, Azezu Asres Nigussie

**Affiliations:** 1 School of Public Health, College of Medicine and Health Sciences, Bahir Dar University, P.O.Box 79, Bahir Dar, Ethiopia; 2 Midwifery Department, College of Medicine and Health Sciences, Bahir Dar University, P.O.Box 79, Bahir Dar, Ethiopia

**Keywords:** School feeding program, Primary school, Student, Nutritional status

## Abstract

**Background:**

Though undernutrition affects academic performance, significant number of Ethiopian school children have undernutrition. To avert nutritional problems the government in collaboration with the world food program has implemented a school feeding program. However, data on the nutritional status of primary school students were scarce in the country. Therefore, this study aimed to assess the relationship between school meals with thinness and stunting among primary school students in Meket Woreda and to identify associated factors.

**Methods:**

A school-based comparative cross-sectional study was conducted among 1091 students, from April 1–27, 2015. The study participants were selected using a multi-stage stratified sampling method. A structured questionnaire was used to collect data. Data entry and analysis were done using Statistical Package for the Social Sciences (SPSS) version 20 software. Descriptive statistics and logistic regression analyses were done. Anthropometric data were analyzed using Anthro-plus software.

**Results:**

In univariate analysis, thinness was commoner in nonschool feeding program schools (37.5%) compared to school feeding program schools (27.8%) but stunting was less common (48.3% versus 58.5%). However, after adjustment for confounding, there was no difference in stunting levels, but the adjusted odds ratio for thinness in nonschool feeding program schools was 2.6 (95% CI: (1.8, 3.8)) times higher than in school feeding program schools. Other independent risk factors for thinness were: having uneducated mother, being a male and taking meal once daily. Independent risk factors for stunting were ethnicity, having uneducated mother, un-piped water supply, taking meal once daily, type of diet and being a male.

**Conclusion:**

The provision of meals seems to offer considerable protection against thinness, though not against stunting. Thus, school meal program should be scaling up into schools in food insecure areas.

## Background

School feeding is the provision of meals to students in class or take-home ration to families with children who attend school regularly [1]. Appropriately designed school feeding intervention improves the nutritional status of school children who suffer from nutrient gaps. It improves cognitive function, school attendance, academic performance, school enrolment and decreases school dropout rates [2–5].

Many developing countries have implemented School Feeding Programs (SFPs) to prevent malnutrition [3, 4]. Because, malnutrition reduces student’s attentiveness, concentration, aptitude, attendance rate, school enrolment and academic performance. All these, in turn, have significant adverse effects on their productivity and quality of life during the adulthood period [5–8]. Not only malnourished but also well-nourished children who are hungry at school encounter difficulties to concentrate and perform complex tasks in the class [9, 10].

According to studies done in Bangladesh, Jamaica, and Kenya, the nutritional status of students who took meals at school was better than students who did not take meals at school [11–14]. However, in Ghana, the nutritional status of students who took meals at school was similar to students who did not take meals at school which was inconsistent with the above study findings [15].

In Ethiopia, undernutrition among school children is a major public health concern. The prevalence of stunting ranges from 41.9% in Arba Minch to17.1%, in Hararghe zone [16, 17]. The magnitude of thinness was 17.9%, in Hararghe zone and 8.0% in Arba Minch [16, 17]. The prevalence of undernutrition was high in the region where this study was conducted. According to a study in Gondar Town 46.1% and 9% of primary school students were stunted and wasted, respectively [18].

In the country, a world food program (WFP) sponsored school feeding program was started in 1994, with an initial pilot project in war-affected zones in the Tigray region. So far, the Ethiopian SFP has provided school meals for students in six regions of the country (Afar, Amhara, Oromiya, Somali, Tigray and Southern Nations and Nationality Peoples Region). In the country, target areas to implement SFP were woredas (the third-level administrative divisions) with chronic food insecurity, lower school enrolment and higher gender disparity [19].

Meket Woreda is one of the food insecure woredas in North Wollo Zone. In the woreda, SFP was implemented in 1995. Due to limited support from the donors, SFP was implemented in five schools with high school dropout rates and transport access. The program provides 120 g fortified blended food with 6 g vegetable oil and 3 g iodized salts. The meal was served for the students in the form of porridge, once a day, on the school days.

Research-based information regarding the nutritional status of Ethiopian children who took meals at school was limited. Thus, this study was designed to assess the relationship between school meals with thinness and stunting among primary school students in Meket Woreda and identify associated factors.

## Methods

### Study area

The study was conducted in Meket Woreda, Ethiopia. According to the 2007 Central Statistics Agency report, the woreda has a total population of 226, 644. From which, 112,246 are females. The woreda was identified as one of the most drought-affected and food insecure woreda in the Amhara Region. According to the woreda education bureau report, in the woreda, there are 50 full-cycle primary schools with 51,869 students. Of these, the SFP was implemented in 5 schools with 5079 students.

### Study design and population

A school-based comparative cross-sectional study was conducted among primary school students, from April 1–27, 2015. All full-cycle primary school students in the woreda were the source population. Students in selected schools during the study period were the study population. In the study area, the school meals were given to the students starting from grade one. Students from grade four and above (students who took school meals three or more years) were included in this study. Similarly, students from grade four and above were also included in schools had no school feeding program. Students who transferred from other schools and not available during the data collection period were excluded from the study.

### Sample size, sampling procedure, and data collection

The required sample size of the study was calculated using two population proportion formulas considering the following assumptions: a 95% confidence level and 80% power, 50% proportion of undernutrition among students who did not take meals at school and 40% proportion of undernutrition among students who took meals at school since there was no previous study. The population allocation ratio is 1:2; the design effect of 2 and 10% non-response rate was added. The final sample size was 1176. Of these, 392 students were from schools had no SFP and 784 students were from schools had SFP.

The study participants were selected using a multi-stage stratified sampling method. Using a list of kebeles (the smallest administrative unite in Ethiopia) as a sampling frame, 12 kebeles were selected from 45 kebeles in the woreda by simple random sampling (SRS) technique (lottery method). Next, schools in selected kebeles were stratified as the SFP schools and non SFP schools.

In selected kebeles, there were 12 schools (8 schools without SFP and 4 schools with SFP). Two schools with SFP and four schools without SFP were selected by the SRS technique using the list of schools in selected kebeles as a sampling frame. The calculated sample size was allocated to the selected schools based on proportion to the size of the students from each school. Finally, using the school registration logbook as a sampling frame, students were selected by the SRS technique. The interview with students’ mothers was conducted in the school compound considering privacy.

Data were collected using the Amharic version (local language) structured interviewer-administered questionnaire. The questionnaire was taken from similar literature [20] and it included socio-demographic characteristics, environmental hygiene, and feeding practice related questions. Feeding practice was assessed using a 24 h recall method. Eight diploma and two BSc nurses were recruited as data collectors and supervisors, respectively.

The weight and height of the students were measured using SECA Germany weighing scales and stadiometers, respectively. During weight measurement, weighing scales were calibrated each day prior to the actual data collection using a known weight material. Weight was measured to the nearest 0.1 kg.. The scale was adjusted before weighing every student by setting it to zero. The students were lightly dressed during having the weight taken.

Height was measured to the nearest 0.1 cm. During taking height, each student stood keeping normal anatomical position without shoes and heels, buttock, shoulder, and back of the head touched measuring board. Then, the headpiece of the measuring board touched the top of the head. For both weight and height, two readings were recorded and the computed averages were used in the analysis.

The z-score values for BMI-for-age and height for age were calculated using WHO Anthro-Plus software. Calculated z-scores of BMI-for-age and height for age were used to classify thinness and stunting using the new WHO 2007 reference value, respectively.

### Operational definition

School feeding program (SFP) participants- are students who took meals at school.

School feeding program (SFP) non- participants- are students who did not take meals at the school.

Height-for-age at ≤ − 3 SD, between-2 SD and > − 3SD, and > − 2 SD of the median value of the WHO international growth reference were defined as severely stunted, stunted and not stunted, respectively.

BMI-for-age at ≤ − 3 SD, between-2 SD and > − 3 SD, between + 1 SD and > − 2 SD, between > + 1 SD and + 2 SD and > + 2 SD of the median value of the WHO international growth reference were defined as severely thin, thinness, normal, overweight and obesity, respectively [21].

### Data quality control

Using trained data collectors and supervisors, pre-testing the questionnaire and checking the weighing scale for functionality were measures taken to assure the quality of data. Moreover, the collected data were reviewed and errors were returned to the data collectors for correction on a daily base. Supervisors and investigators closely supervised the data collection procedure.

### Data processing and analysis

Data were entered and analyzed using SPSS version 20 software. The nutritional status of the students was compared. Bivariate and multivariable logistic regressions were done to identify factors associated with stunting and thinness. Hosmer-Lemeshow Goodness-of-fit-test was done to check model fitness. Correlation between independent variables was checked using the Pearson Correlation Coefficient. The model was built with backward elimination. The crude odds ratio was done and *p*-value ≤ 0.2 was taken as a cut-off point to select variables for multivariate analysis. Age was controlled during the multivariable logistic regression analysis. The adjusted Odds ratio was computed to determine the strength of association and control confounders. The *p*-value of less than 0.05 was considered statistically significant.

### Ethical approval and consent to participate

The study was approved by the Ethical Review Board of Bahir Dar University. A letter of permission was taken from zonal and woreda health bureaus as well as the school administrators. Since it causes less than minimum risk, the ethical committee approved to take verbal consent. Based on this, verbal consent was taken from parents and assent was taken from students. Privacy and confidentiality were maintained throughout the study period by excluding personal identifiers from the data collection tools.

## Results

### Socio-demographic characteristics of the students and their parents

A total of 1091 primary school students were recruited in the study, with a response rate of 92.8%. Of which 65.8% (*n* = 718) of them were from schools had SFP and 34.2% (*n* = 373) of the study participants were from schools had no SFP. The mean (+SD) age of the respondents from schools had SFP and had no SFP were 12.12 (+ 1.36) years and 12.73 (+ 1.46) years, respectively (Table 1).

**Table 1:** Socio-demographic characteristics of the primary school students and their parents, Meket Woreda, April 2015 (*n* = 1091)

Variables	SFP schools = 718	Non SFP schools = 373	***P***-value
**Age in years**			
10–12	476 (66.3)	174 (46.7)	0.001
13–14	200 (27.9)	153 (41.0)	
> 15	42 (5.8)	46 (12.3)	
**Sex**			
Male	364 (50.7)	176 (47.2)	0.30
Female	354 (49.3)	197 (52.8)	
**Religion**			
Orthodox	686 (95.5)	369 (98.9)	0.005
Muslim	32 (4.5)	4 (1.1)	
**Ethnicity**			
Amhara	700 (97.5)	370 (99.2)	0.87
Agew	18 (2.5)	3 (0.8)	
**Mother’s Education**			
Have no formal education	636 (88.6)	343 (92.0)	0.10
Have formal education	82 (11.4)	30 (8.0)	
**Father’s Education**			
Have no formal education	626 (87.2)	241 (64.6)	0.001
Have formal education	92 (12.8)	132 (35.4)	
**Mother’s occupation**			
Housewife	640 (89.1)	266 (71.3)	0.001
Daily laborer	38 (5.3)	6 (1.6)	
Petty trader	40 (5.6)	101 (27.1)	
**Father’s occupation**			
Farmer	578 (80.5)	336 (90.1)	0.001
Petty trader	94 (13.1)	30 (8.0)	
Daily laborer	46 (6.4)	7 (1.9)	
**Marital status of their mothers**			
Married	638 (88.9)	333 (89.3)	0.96
Divorced	72 (10.0)	30 (8.0)	
Never married	8 (1.1)	10 (2.7)	
**Currently living with**			
Both biological parents	582 (81.1)	301 (80.7)	0.65
Their mothers	84 (11.7)	49 (13.1)	
Their grand parents	52 (7.2)	23 (6.2)	
**Residence**			
Urban	72 (10.0)	31 (8.3)	0.417
Rural	646 (90.0)	342 (91.7)	
**Family size**			
< 5	250 (34.8)	84 (22.5)	0.001
5–6	320 (44.6)	215 (57.6)	
> 6	148 (20.6)	74 (19.9)	
**Possession of Television**			
Yes	90 (12.5)	8 (2.1)	< 0.001
No	628 (87.5)	365 (97.9)	
**Possession of Radio**			
Yes	136 (18.9)	31 (8.3)	< 0.001
No	582 (81.1)	342 (91.7)	

Have no formal education = respondents who did not attained any schooling, have formal education = respondent attained primary and above education, Petty trader = were respondents who sell goods on the street.

The majority, 686(95.5%) of respondents from schools had SFP and 369(98.9%) of students from schools had no SFP were orthodox Christian followers. Almost all, 700(97.5%) of the study participants from schools had SFP and 370(99.2%) of students from schools had no SFP were Amhara in their ethnicity. In both groups, 81 % of students live with their both biological parents. Regarding their mothers, 636(88.6%) of students’ mothers from schools had SFP and 343(92.0%) of students’ mothers from schools without SFP had no formal education (Table 1).

### Dietary intake, hygiene and sanitation of the primary school students

The proportions of students who took more than one meals (97.8% Vs 94.4%) and wash their hands with soap (30.9% Vs 14.5%) were higher among SFP schools compared to non SFP schools. The number of students who took other foodstuffs with Shero was higher in non SFP schools 253(67.8%) compared to SFP schools 126(17.5%). The proportion of students who used latrine (91.2% Vs 57.9%) and drunk pipe/protected spring water (49.3%Vs 10.6%) were also higher in non SFP schools than SFP schools (Table 2).

**Table 2:** Dietary intake, hygiene and sanitation of the primary school students, Meket Woreda, April 2015 (*n* = 1091)

Variables	SFP schools = 718	Non SFP schools = 373	***P***-value
**Frequency of food intake at home per 24 h**			
1times	16 (2.2)	21 (5.6)	0.006
> 1times	702 (97.8)	352 (94.4)	
**Frequent diet the child take at home**			
Shero	592 (82.5)	120 (32.2)	< 0.001
Shero, kik, meat, vegetable and milk	126 (17.5)	253 (67.8)	
**Source of drinking water**			
River/spring	642 (89.4)	189 (50,7)	< 0.001
Pipe/protected spring	76 (10.6)	184 (49.3)	
**Use latrine**			
Yes	416 (57.9)	340 (91.2)	< 0.001
No	302 (42.1)	33 (8.8)	
**The child wash his/her hands with soap before eating**			
Yes	222 (30.9)	54 (14.5)	< 0.001
No	496 (69.1)	319 (85.5)	

*Shero* Stew made from legume flour, *kik* Split legumes stew.

### Nutritional status of primary school students

The prevalence of thinness was higher among students who did not take meals at school 140(37.6%) than students who took meals at school 200(27.8%). The proportions of students with severe thinness were 70(9.7%) and 32(8.6%) in schools having SFP and in schools did not have SFP, respectively. On the other hand, the magnitude of stunting was higher among students in schools having SFP 420(58.5%) compared with students in schools did not have SFP 180 (48.3%). The prevalence of severely stunted students was 160(22.3%) among students who took meals at school compared with students who did not take meals at school 68(18.2%).

### Factors associated with stunting in primary school children

In the bivariate logistic regression analysis not taking a meal at school, having a mother with no formal education, living in a household did not use a latrine, taking Shero at home frequently, drinking river or spring water, taking a meal once per day at home, being in Agew ethnic group and males were statistically associated with stunting (Table 3).

**Table 3:** Factors associated with stunting among the primary school children, Meket Woreda, 2015

Variables	Stunting		COR (95%CI)	AOR (95%CI)
	Stunted(*n* = 600)	Not stunted (*n* = 491)		
**Ethnicity**				
Amhara	583 (97.2)	487 (99.2)	1.00	1.00
Agew	17 (2.8)	4 (0.8)	3.5 (1.1,10.6)	3.9 (1.2,12.3)
**Educational status of the mother**				
Have no formal education	555 (92.5)	424 (86.4)	1.9 (1.3,2.9)	2.1 (1.4,3.3)
Have formal education	45 (7.5)	67 (13.6)	1.00	1.00
**Drinking water**				
Pipe or protected	117 (19.5)	143 (29.1)	1.00	1.00
River and unprotected spring	483 (80.5)	348 (70.9)	1.6 (1.2,2.2)	1.5 (1.1,2.1)
**Frequency of food intake at home per 24 h**				
1 times	28 (4.7)	9 (1.8)	2.6 (1.2,5.6)	2.6 (1.2,5.9)
> =2 times	572 (95.3)	482 (98.2)	1.00	1.00
**Take meals at school**				
Yes	420 (70.0)	298 (60.7)	1.5 (1.1,1.9)	
No	180 (30.0)	193 (39.3)	1.00	
**Sex of students**				
Male	335 (55.8)	205 (41.8)	1.7 (1.3,2.2)	1.6 (1.3,2.1)
Female	265 (44.2)	286 (58.2)	1.00	1.00
**Family use latrine**				
Yes	391 (65.2)	365 (74.3)	1.00	
No	209 (34.8)	126 (25.7)	1.5 (1.1,2.0)	
**Frequent diet the child take at home**				
Shero	431 (71.8)	281 (57.2)	1.9 (1.4,2.4)	1.7 (1.3,2.3)
Shero, kik, meat, vegetable and milk	169 (28.2)	210 (42.8)	1.00	1.00

*AOR* Adjusted Odds Ratio, *COR* Crude Odds Ratio, ***95%****CI* 95 % confidence interval, Shero Stew made from legume flour, *kik* Split legumes stew.

After controlling age, in the multivariable logistic regression analysis, taking meals at school was not associated with stunting. Being in the Agew ethnic group, taking Shero at home frequently, having a mother with no formal education, being male, drinking river or spring water and taking a meal once daily at home had a statistically significant association with stunting (Table 3).

### Factors associated with thinness in primary school children

In the bivariate logistic regression analysis having a mother and father with no formal education, being a male, taking Shero frequently at home, living in the household didn’t have radio and television, having married and housewife mother, being a rural residence, taking a meal once daily at home, students who did not take meals at school and wash their hands before eating were statistically associated with thinness (Table 4).

**Table 4:** factors associated with thinness of the primary school children, Meket Woreda, 2015

Variables	Thinness		COR (95%CI)	AOR (95%CI)
	Thin(***n*** = 340)	Not thin (***n*** = 751)		
**Take meals at school**				
Yes	200 (58.8)	518 (69.0)	1.00	1.00
No	140 (41.2)	233 (31.0)	1.5 (1.1,2.0)	2.6 (1.8,3.8)
**Educational status of the mother**				
Have no formal education	334 (98.2)	645 (85.9)	9.1 (3.9,21.0)	5.3 (2.2,12.6)
Have formal education	6 (1.8)	106 (14.1)	1.00	1.00
**Possession of television**				
Yes	10 (2.9)	88 (11.7)	1.00	
No	330 (97.1)	663 (88.3)	4.3 (2.2,8.5)	
**Possession of radio**				
Yes	31 (9.1)	136 (18.1)	1.00	1.00
No	309 (90.9)	615 (81.9)	2.2 (1.4,3.3)	1.7 (1.1,2.7)
**Sex of students**				
Male	208 (61.2)	332 (44.2)	1.9 (1.5,2.5)	1.9 (1.4,2.5)
Female	132 (38.8)	419 (55.8)	1.00	1.00
**Educational status of the father**				
Have no formal education	294 (86.5)	573 (76.3)	1.9 (1.3,2.8)	
Have formal education	46 (13.5)	178 (23.7)	1.00	
**Occupational status of the mother**				
Housewife	330 (97.1)	646 (86.0)	5.3 (2.7,10.3	3.1 (1.5,6.4)
Daily laborer and petty trader	10 (2.9)	105 (14.0)	1.00	
**Frequent diet the child take at home**				
Shero	255 (75.0)	457 (60.9)	1.9 (1.4,2.5)	2.7 (1.6,3.9)
Shero, kik, meat, vegetable and milk	85 (25.0)	294 (39.1)	1.00	1.00
**Frequency of food intake at home /24 h**				
1 times	23 (6.8)	14 (1.9)	3.8 (1.9,7.5)	3.5 (1.6,7.6)
-- > =2 times	317 (93.2)	737 (98.1)	1.00	1.00
**The child wash his/her hand with soap before eating**				
Yes	60 (17.6)	216 (28.8)	1.00	
No	280 (82.4)	535 (71.2)	1.8 (1.3,2.5)	
**Marital status of the mother**				
Married	314 (92.4)	657 (87.5)	1.7 (1.1,2.7)	
Divorced, never married	26 (7.6)	94 (12.5)	1.00	
**Residence**				
Urban	21 (6.2)	82 (10.9)	1.00	
Rural	319 (93.8)	669 (89.1)	1.8 (1.1,3.0)	

*AOR* Adjusted Odds Ratio, *COR* Crude Odds Ratio, ***95%****CI* 95 % confidence interval.

After controlling for age, in the multivariable logistic regression analysis students who did not take meals at school were 2.6 times at a higher risk to develop thinness than their counterparts [AOR = 2.6, 95% CI: (1.8, 3.8)]. Having mother had no formal education, being male, taking a meal once a day at home, taking Shero frequently at home, living in a household have no radio and having a housewife mother were independent predictors of thinness (Table 4).

## Discussion

In this study, the prevalence of thinness was higher among students who did not take meals at school than students who took meals at school. The positive significant association between taking meals at school and thinness persists after adjusting for the potential confounders. Since thinness is a nutritional problem due to the current imbalance between nutrient intake and requirement, the association between school meal and thinness persists after adjustment for the potential confounders. This finding was similar to the study findings in Western Kenya and Ghana [22–24]. The possible explanation is that students who took meals at school might have a higher probability of meeting their nutrient requirements than students who did not take meals at school.

On the other hand, this finding was not consistent with the study finding in Ghana [20]. This discrepancy might be due to the difference in the quality and quantity of school meals. Moreover, this could be explained by the difference in socioeconomic status and nutrition knowledge of Ethiopian and Gahanna parents.

Though it wasn’t significant after adjusting for the potential confounders, the prevalence of stunting was higher among students who took meals at school compared with students who did not take meals at school. This finding was similar to the study finding in Ghana [20]. This might be due to the reason that students who participated in the SFP were more likely to be stunted starting from the first thousand days of life. The provision of nutrition supplements at school beyond the period of maximum growth may not be effective to recover from growth faltering because school age may be too old to experience catch-up growth.

This finding was not in agreement with the study finding in Western Kenya and Ghana [22, 23]. This discrepancy might be due to differences in enrolment criteria of school feeding programs between Ethiopia and other countries. The quality and quantity of school meals may also differ between countries. Furthermore, it might be due to the difference in home background, nutrition, and child care practice of parents between Ethiopia and other countries.

Low socioeconomic status was a risk factor for both stunting and thinness among primary school students. This might be due to reduced availability and accessibility of foodstuffs in the household of poor people. This, in turn, affects the nutrient intake of school children. Students who had a housewife mothers were more likely to be thin than students who had daily laborer or petty trader mother. This finding was supported by the study findings in central India and Okolobiri [25, 26]. The possible explanation might be working mothers are more likely to have income and decision making power to purchase health service and food items.

The likelihood of both stunting and thinness was higher among boys compared to girls. Similar findings were reported from Jamaica, India, and Sari Lanka [7, 9, 25]. The reason might be due to the high nutrient requirement of boys than girls. Moreover, in Ethiopia, male students are more likely to participate in cattle keeping and other farm activities which increased energy expenditure than females. Males stay longer out of home which reduces the opportunity of taking a meal during the day.

The odds of having thinness was higher among the students whose family had no radio compared to their counterparts. This finding was supported by the previous study finding in Ethiopia [27]. Families who had radio were more likely to get nutrition and child care related information through media than those who did not have a radio.

Students who had uneducated mothers and frequently took only Shero at home were more likely to develop stunting and thinness than their counterparts. This finding was supported by the study findings in Ethiopia, Egypt, Kenya, Bangladesh, and India [11, 14, 25, 28, 29]. In Egypt and Kenya [13, 28] students who took variety of foods were less likely to develop undernutrition. The reason for this might be the likelihood of students who took variety of foods to meet nutrient requirements than students who regularly take monotonous diets. Educated mothers have a higher probability of being knowledgeable about feeding practice and health-seeking behavior. In this study, students in Agew ethnic group were more likely to be stunted than students in the Amhara ethnic group. This might be due to the poor socioeconomic status of their parents.

Strength and limitation: Comparative nature of this study among students who took meals at school and who did not take a meal at school could be the strength of this study. However, the following limitations should be considered during interpreting the results. Though we have tried to control for potential confounders there may be residual confounders from unmeasured variables. The content of school meals and the effect of school meals on school attendance were not assessed in this study.

## Conclusion

The provision of meals seems to offer considerable protection against thinness, though not against stunting. This may justify scaling up school meal programs in food insecure areas. Further studies should be conducted starting from the window of opportunity and to assess nutrient content of school meals and its effect on school attendance.

## Data Availability

The datasets analyzed during the current study are available from the corresponding author on reasonable request.

## Abbreviations

AOR: Adjusted Odds Ratio
BSC: Bachelor Science
BMI: Body Mass Index
COR: Crude Odds Ratio
SFP: School Feeding Program
WFP: World Food Program
